# Prone position and recruitment manoeuvre: the combined effect improves oxygenation

**DOI:** 10.1186/cc10235

**Published:** 2011-05-16

**Authors:** Gilles Rival, Cyrille Patry, Nathalie Floret, Jean Christophe Navellou, Evelyne Belle, Gilles Capellier

**Affiliations:** 1 Service de pneumologie, Centre Hospitalier Régional et Universitaire de Besançon, 3 Bd Fleming, Besançon F-25000, France; 2 Service de réanimation médicale, Centre Hospitalier Régional et Universitaire de Besançon, 3 Bd Fleming, Besançon F-25000, France; 3 Département d'informatique médicale, Centre Hospitalier Régional et Universitaire Besançon, 3 Bd Fleming, Besançon F-25000, France; 4 Equipe d'accueil EA 3920, Unité de Formation et de Recherche Médecine Pharmacie, Université de Franche Comté, 19 rue Ambroise Paré, les Hauts du Chazal Besançon F-25000 France

## Abstract

**Introduction:**

Among the various methods for improving oxygenation while decreasing the risk of ventilation-induced lung injury in patients with acute respiratory distress syndrome (ARDS), a ventilation strategy combining prone position (PP) and recruitment manoeuvres (RMs) can be practiced. We studied the effects on oxygenation of both RM and PP applied in early ARDS patients.

**Methods:**

We conducted a prospective study. Sixteen consecutive patients with early ARDS fulfilling our criteria (ratio of arterial oxygen partial pressure to fraction of inspired oxygen (PaO_2_/FiO_2_) 98.3 ± 28 mmHg; positive end expiratory pressure, 10.7 ± 2.8 cmH_2_O) were analysed. Each patient was ventilated in both the supine position (SP) and the PP (six hours in each position). A 45 cmH_2_O extended sigh in pressure control mode was performed at the beginning of SP (RM1), one hour after turning to the PP (RM2) and at the end of the six-hour PP period (RM3).

**Results:**

The mean arterial oxygen partial pressure (PaO_2_) changes after RM1, RM2 and RM3 were 9.6%, 15% and 19%, respectively. The PaO_2 _improvement after a single RM was significant after RM3 only (*P *< 0.05). Improvements in PaO_2 _level and PaO_2_/FiO_2 _ratio were transient in SP but durable during PP. PaO_2_/FiO_2 _ratio peaked at 218 mmHg after RM3. PaO_2_/FiO_2 _changes were significant only after RM3 and in the pulmonary ARDS group (*P *= 0.008). This global strategy had a benefit with regard to oxygenation: PaO_2_/FiO_2 _ratio increased from 98.3 mmHg to 165.6 mmHg 13 hours later at the end of the study (*P *< 0.05). Plateau airway pressures decreased after each RM and over the entire PP period and significantly after RM3 (*P *= 0.02). Some reversible side effects such as significant blood arterial pressure variations were found when extended sighs were performed.

**Conclusions:**

In our study, interventions such as a 45 cmH_2_O extended sigh during PP resulted in marked oxygenation improvement. Combined RM and PP led to the highest increase in PaO_2_/FiO_2 _ratio without major clinical side effects.

## Introduction

Acute respiratory failure is a common pathology in intensive care units. Management of acute respiratory distress syndrome (ARDS) and acute lung injury (ALI) [1] remains a problem. Life care support such as mechanical ventilation is used to maintain or improve oxygenation. Nevertheless, as is true of many therapies, side effects such as ventilation-induced lung injury (VILI) and oxygen toxicity have been described [2,3]. Moreover, increased mortality in ARDS patients is well established when patients are ventilated with high tidal volume (*V*_t_) and high plateau pressure. Nowadays, low *V*_t _and limited plateau pressure below 30 cmH_2_O have been associated with lower mortality and less inflammation [4-6]. Mechanical ventilation is therefore recommended as a lung-protective strategy. However, such ventilator settings are reported to induce hypoxemia, hypercapnia, alveolar derecruitment and atelectasis, which also contribute to lung injury [7,8]. Inflated, normal, poorly aerated or nonaerated airway spaces coexist, and ventilation may induce (1) shear stress at the boundaries of these spaces, (2) inadequate cyclic opening and (3) closing of alveoli. Inflammation as well as cellular and epithelial damage may be associated with this type of ventilation [9,10]. The "open lung concept" was developed to fight against these ventilatory side effects and to improve oxygenation [11-16]. Opening pressures used should recruit poorly aerated or nonaerated airway spaces, and once this procedure is carried out, positive end expiratory pressure (PEEP) can be applied to stabilize cyclic opening and closing of alveoli to decrease VILI and to maintain oxygenation improvement [17-21]. To reinforce this strategy, an animal study suggested that a low stretch/open lung strategy compared to a low stretch/rest lung strategy was associated with lower mortality, decreased inflammatory response, more apoptosis and less epithelial damage [22]. Prone position (PP) [23-26] and recruitment manoeuvre (RM) [27-36] have been studied, and some benefit on alveolar recruitment, VILI and oxygenation has been demonstrated [37]. In daily practice and from a practical point of view, lung-protective ventilation is recommended. In addition to this strategy, RM can be performed while patients are in supine position (SP), and they can be turned to PP if hypoxemia remains a concern. In the present study, we tested the hypothesis that RM might have a different impact on oxygenation according to whether it was performed with patient in SP or in early or late PP. We therefore conducted a prospective study to evaluate the benefits of extended sigh using 45 cmH_2_O airway pressure combined with PP in acute respiratory failure.

## Materials and methods

### Population

From June 2002 to March 2003, we prospectively studied, during the first week of ventilation, patients with ARDS or ALI, defined according to the criteria of the ARDS American European Consensus Conference [1]. This study was approved by our local hospital ethics committee (Comité d'éthique clinique du CHU de Besançon). Written informed consent was waived. Patients were sedated, paralysed and ventilated in the volume control mode. Vasopressive drugs and fluid resuscitation were used as required to obtain a mean arterial pressure (MAP) of 75 mmHg. Patients with uncontrolled low cardiac output, a temporary pacemaker, bronchospasm or barotrauma were excluded.

### Basic ventilation

A lung-protective ventilation strategy was used to maintain plateau pressure below 30 cmH_2_O [20]. PEEP was adjusted to obtain 92% ± 2% oxygen saturation measured via pulse oximetry (SpO_2_) with fraction of inspired oxygen (FiO_2_) between 60% and 80%. PEEP may have been increased to 6, 8, 10, 12 or 14 cmH_2_O to achieve the above criteria. Once these FiO_2 _and SpO_2 _criteria had been reached, ventilatory parameters were not changed. If FiO_2 _was still higher than 80% with a PEEP of 14 cmH_2_O, the increase in PEEP was interrupted and the patient was included in the study at that time. The inspiratory/expiratory (I/E) ratio was adjusted between 1:2 and 1:3. Basic ventilation was used, except when RM was performed. Mount connections were systematically removed. Heat humidifiers were used.

### Recruitment manoeuvre

**Figure 1:**
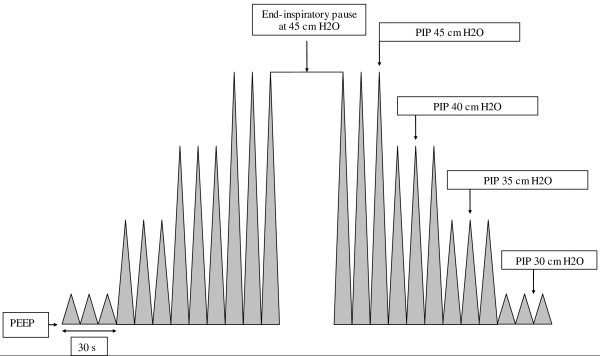
**Recruitment maneuver in pressure control mode ventilation**.

The RM consisted of changing the ventilatory mode to the pressure control mode and increasing pressure levels every 30 seconds to successively obtain 35, 40 and 45 cmH_2_O peak inspiratory pressures (PIP) (Figure 1). Once the 45 cmH_2_O PIP had been reached, a 30-second end-inspiratory pause was performed using the inspiratory pause function. The I/E ratio was maintained at 1:1 during RM. Respiratory frequency, PEEP and FiO_2 _were similar during RM. We returned to basic ventilation every 30 seconds throughout the various 30-second steps described above. At the end of the RM, previous ventilatory adjustments were applied.

### Prone position

PP was maintained for six hours. FiO_2 _may have been temporarily increased to 100% while the patient was turned, and then it decreased back to the initial FiO_2 _level.

### Protocol

**Figure 2:**
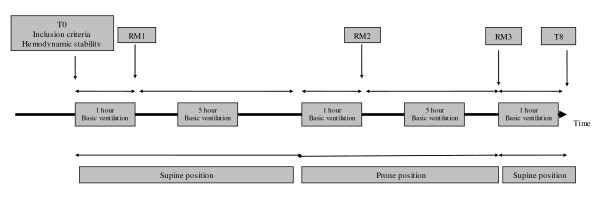
**Study design**. RM, recruitment manoeuvre; PEEP, positive end expiratory pressure, PIP, peak inspiratory pressure.

Two six-hour periods were used: one with patient in SP and one in PP. The first RM was performed at the beginning of SP (one hour after stabilization), the second one was performed one hour after turning the patient to PP and the last one was performed at the end of PP (Figure 2). Ventilatory settings, gas exchanges and haemodynamic parameters were recorded each time (from time 0 to time 8) in SP and PP: at the time of inclusion, before and immediately after each RM, before PP and one hour after turning the patient to SP.

### Statistical methods

For this descriptive and analytical study, nonparametric tests were used. The Wilcoxon paired test was carried out to compare the variables before and after recruitment manoeuvres. If the number of equal variables was high, a sign test was implemented. The quantitative variables studied are reported in the tables as means ± standard deviations. A *P *value < 0.05 was considered statistically significant. The different analyses were carried out by using SYSTAT 8.0 software.

## Results

### Population

Table 1 shows the patient demographics. Sixteen ARDS patients were prospectively included, 12 with pulmonary ARDS and four with extrapulmonary ARDS. Thirteen patients completed the study, while for three patients the protocol was interrupted at some point. Pneumonia and pancreatitis were the main causes of ARDS. The patients were 63 years old on average. The mean Simplified Acute Physiology Score II was 44.7. The mean number of organ failures was about two. The mortality rate was 43.7%. Seven patients died, five as a result of pulmonary ARDS and two as a result of extrapulmonary ARDS.

**Table 1:** Patient population^a^

Patient demographics	Pulmonary ARDS	Extrapulmonary ARDS
Number of patients	12	4
Average age, years	63	66
SAPS II	47	39
Organ failure^b^	2.5	1.75
PaO_2_/FiO_2 _ratio at time 0, mmHg	99	97.5
Deaths, *n*	5	2
Diagnosis, *n*		
Pneumonia	9	
Aspiration	3	
Acute pancreatitis		4

^a^ARDS, acute respiratory distress syndrome; SAPS II, Simplified Acute Physiology Score II; PaO_2_/FiO_2 _ratio, ratio of arterial oxygen partial pressure to fraction of inspired oxygen. ^b^Organ Dysfunction and/or Infection score was used to quantify the number of organ failures.

### Ventilatory settings

Table 2 shows the ventilator settings maintained throughout the whole study and their different effects on peak and plateau airway pressure. These decreased after each RM and over the entire PP period. The decrease in plateau pressure was significant after RM3 (*P *= 0.02). Plateau pressures at time 8 were lower than T0, but the decrease was not statistically significant.

**Table 2:** Ventilatory settings used during the study^a^

	SP				PP				SP
			
Ventilatory setting	Time 0	Time 1 (RM1) time 2		Time 3	Time 4 (RM2) time 5		Time 6 (RM3) time 7		Time 8
*V*_t_, mL	536 ± 105	522 ± 106.8	534 ± 102	532 ± 102	511 ± 99	511 ± 98.7	512 ± 97.8	512 ± 98.2	512 ± 98
RR, breaths/minute	19 ± 4.1	19.5 ± 4.1	19.5 ± 4.3	19.5 ± 4.3	20 ± 4.4	20 ± 4.4	20 ± 4.4	20 ± 4.4	20 ± 4.4
V^°^, L/minute	10.5 ± 2.3	10.2 ± 2	10.4 ± 2.2	10.4 ± 2.1	10.2 ± 2.2	10.2 ± 2.2	10.2 ± 2.2	10.3 ± 2.2	10.3 ± 2.2
External PEEP, cmH_2_O	9.8 ± 2.8	9.8 ± 2.8	9.8 ± 2.8	9.8 ± 2.8	10.1 ± 2.6	10.1 ± 2.6	10.1 ± 2.6	10.1 ± 2.6	10.3 ± 2.7
Total PEEP, cmH_2_O	10.7 ± 2.8	10.6 ± 2.8	10.8 ± 2.9	10.8 ± 2.7	10.9 ± 3	11.4 ± 3.3	10.5 ± 2.8	10.6 ± 2.9	10.8 ± 3
Paw, cmH_2_O	31.7 ± 4.7	30.5 ± 6	30.2 ± 5.7	31 ± 4.9	29 ± 5.2	30.5 ± 5.2	29 ± 5.9	28 ± 5.3	29 ± 5.3
Pplat, cmH_2_O	24.6 ± 5.8	24.5 ± 5.7	24 ± 5.5	25.3 ± 5^b^	24.2 ± 4.6	24 ± 4.1	23.4 ± 4.9	22.7 ± 5^c^	23 ± 5.1

^a^Paw: peak airway pressure; Pplat: plateau pressure; *V*_t_: tidal volume; RR: respiratory rate; V°: minute volume; PEEP: positive end expiratory pressure; SP: supine position; PP: prone position; RM recruitment maneuver. Ventilatory settings were measured each time (from time 0 to time 8) in SP and PP (see Figure 2): inclusion, before and after each RM, before PP, and at the end of the protocol (1 hour after turning to the SP). ^b^Time 3 versus time 2: *P *= 0.035; ^c^time 6 versus time 7: *P *= 0.02. All data are expressed as means ± standard deviations.

### Gas exchange

Table 3 shows the effects of gas exchange.

**Table 3:** Gas exchanges used during the study^a^

	SP				PP				SP
			
Gas exchanges	Time 0	Time 1 (RM1) time 2		Time 3	Time 4 (RM2) time 5		Time 6 (RM3) time 7		Time 8
pH	7.37 ± 0.08	7.37 ± 0.07	7.40 ± 0.08^b^	7.36 ± 0.08^c^	7.39 ± 0.08	7.43 ± 0.08^d^	7.40 ± 0.09	7.47 ± 0.08^e^	7.40 ± 0.08^f^
PaO_2_, mmHg	75.6 ± 19	85.4 ± 28	94.5 ± 39	88.9 ± 24	117 ± 63	138 ± 77	138.6 ± 70	171.5 ± 84^g^	129.5 ± 66^h^
PaCO_2_, mmHg	39 ± 7	39 ± 7.7	35 ± 7.4^i^	40 ± 8.4^j^	37 ± 8.4	35 ± 7.7^k^	36.4 ± 8.4	31.5 ± 8.4^l^	36.4 ± 7.3^m^
PaO_2_/FiO_2 _ratio, mmHg	98.3 ± 28	111.4 ± 41.2	123 ± 52.3	115.5 ± 36	151.2 ± 75.7	178 ± 99	177 ± 75	218.2 ± 99.5^n^	165.6 ± 84.5°

^a^SP: supine position; PP: prone position; RM: recruitment maneuver; PaO_2_: arterial oxygen partial pressure; PaCO_2_: arterial carbon dioxide partial pressure; PaO_2_/FiO_2 _ratio, ratio of arterial oxygen partial pressure to fraction of inspired oxygen. Gas exchanges were measured each time (from time 0 to time 8) in SP and PP (see Figure 2): inclusion, before and after each RM, before PP and at the end of the protocol (1 hour after turning to the SP). ^b^pH time 2 versus time 1, *P *≤ 0.001; ^c^pH time 3 versus time 2, *P *≤ 0.05; ^d^pH time 5 versus time 4, *P *≤ 0.001; ^e^pH time 7 versus time 6, *P *≤ 0.05; ^f^pH time 8 versus time 7, *P *≤ 0.01; ^g^PaO_2 _time 7 versus time 6, *P *≤ 0.05; ^h^PaO_2 _time 8 versus time 0, *P *≤ 0.05; ^i^PaCO_2 _time 2 versus time 1, *P *≤ 0.05; ^j^PaCO_2 _time 3 versus time 2, *P *≤ 0.05; ^k^PaCO_2 _time 5 versus time 4, *P *≤ 0.05); ^l^PaCO_2 _time 7 versus time 6, *P *≤ 0.05; ^m^PaCO_2 _time 8 versus time 7, *P *≤ 0.01; ^n^PaO_2_/FiO_2 _ratio time 7 versus time 6, *P *≤ 0.05; °PaO_2_/FiO_2 _ratio time 8 versus time 0, *P *≤ 0.05. All data are expressed as means ± standard deviations.

#### Impact of RM on gas exchange

PaO_2 _and PaO_2_/FiO_2 _ratio increased after each RM. The mean PaO_2 _changes before and after RM1, RM2 and RM3 were 9.6%, 15% and 19%, respectively. The PaO_2_/FiO_2 _ratio peaked at 218 mmHg after RM3. The improvement before and after a single RM was significant after RM3 only (*P *< 0.05). Arterial carbon dioxide partial pressure (PaCO_2_) decreased after each RM (*P *< 0.05).

#### Impact of RM on gas exchange depending on body position

Improvements in PaO_2 _and PaO_2_/FiO_2 _ratio were transient in SP but durable during PP between RM2 and RM3. The decrease in PaCO_2 _after RM1 was transient in SP and durable in PP.

#### Impact of the global strategy on gas exchange

When patients were included, the PaO_2_/FiO_2 _ratio was 98.3 mmHg with 79% FiO_2 _and 10 cmH_2_O PEEP. At the end of the study, in SP and compared to the beginning, the PaO_2_/FiO_2 _ratio was significantly higher at 165.6 mmHg (*P *< 0.05). PaCO_2 _decreased from 39 mmHg at the beginning of the study to 36.4 mmHg at the end of the study.

#### Impact of RM on gas exchange depending on extrapulmonary or pulmonary ARDS

In the pulmonary ARDS group, the PaO_2_/FiO_2 _ratio improved from 115 ± 47 mmHg to 128 ± 59 mmHg after RM1, from 162 ± 83 mmHg to 196 ± 104 mmHg after RM2 and from 185 ± 83 mmHg to 230 ± 101 mmHg after RM3. In patients with extrapulmonary ARDS, the PaO_2_/FiO_2 _ratio improved from 102 ± 19 mmHg to 107 ± 22 mmHg after RM1, from 113 ± 12 mmHg to 112 ± 35 mmHg after RM2 and from 149 ± 23 mmHg to 154 ± 78 mmHg after RM3. In these subgroups, changes in PaO_2_/FiO_2 _ratio were significant only after RM3 and only in the pulmonary ARDS group (*P *= 0.008).

### Haemodynamics

**Figure 3:**
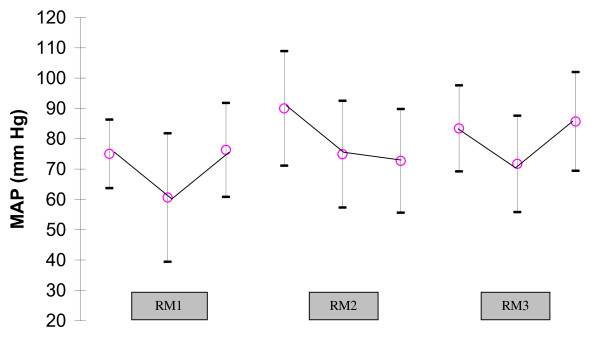
**Changes in mean arterial pressure (MAP) during the three recruitment maneuvers showing significant decrease in MAP**. RM1: *P *= 0.008; RM2: *P *= 0.03; RM3: *P *= 0.01.

Figure 3 shows the haemodynamic effects. Vasopressive drug infusion rates were not modified throughout the entire study. A significant decrease in MAP was found when extended sighs were performed. However, they were reversible when the manoeuvre was stopped.

### Complications

One patient had reversible bronchoconstriction after an extended sigh. PP could not be performed in a second patient because of heart rate disorders. PP had to be interrupted in the first few minutes for a third patient because of major desaturation related to an increase in airway pressure (above 50 cmH_2_O) due to abdominal compartment syndrome. RM did not cause pulmonary barotrauma. Predominant dermabrasions on the chest and the abdomen as well as facial oedema were observed after PP in four patients.

## Discussion

The main findings of our early ARDS/ALI study are that there are probable combined effects of RM and PP as well as a larger PaO_2 _improvement when RM is performed while the patient is in PP and probably after an extended period of time.

RMs have been proved to be efficient to protect the lung while improving oxygenation [37,38]; however, a computed tomography-based study performed during RM in an animal model indicated that there were no protective effects against hyperinflation because of persistent lung inhomogeneity during the RM procedure [39]. A recent PP meta-analysis suggested a positive result on oxygenation and mortality and that VILI may be reduced or delayed during PP [37,40,41]. The combination of PP and RM may be a safe strategy to use for improvement of oxygenation and to avoid VILI. However, this strategy has not been studied often in the setting of acute respiratory failure [42-45].

In an oleic acid-induced lung injury model, Cakar *et al*. [42] studied the combination of PP and a 60 cmH_2_O sustained inflation over 30 seconds. These authors observed greater oxygen improvement with reduced alveolar stress when PP was used. Three clinical studies in humans have tested the benefits of such a strategy. The findings of those studies are summarized in Table 4.

**Table 4:** Summary of studies^a^

		Baseline ventilation					Best PaO_2_/FiO_2 _ratio variation (mmHg), PP + RM			
					
Study	ARDS type, number of patients	*V*_t_, mL	RR, breaths/minute	PEEP, cmH_2_O	PaO_2_/FiO_2 _ratio, mmHg	Pplat, cmH_2_O	Pre-PaO_2_/FiO_2 _ratio, mmHg	Post- PaO_2_/FiO_2 _ratio, mmHg	RM type	Study design
Pelosi *et al*., 2003 [43]	Early ARDS (*n *= 10): 6 pulmonary, 4 extrapulmonary	About 7 mL/kg 590 mL	14	14	121	32	193	240	Sigh: Three consecutive volume-limited breaths/minute with a plateau pressure of 45 cmH_2_O	Following period of the study: 2-hour baseline SP 1-hour sigh SP 1-hour baseline SP 2-hour baseline PP 1-hour sigh PP 1-hour baseline PP Measurements taken at end of each period
Lim *et al*., 2003 [45]	Early ARDS (*n *= 47): 37 pulmonary, 10 extrapulmonary 19 patients from a preliminary study	About 8 mL/kg	20	10	128	-	166	200	Extended sigh Inflation phase: PEEP was increased by 5 cmH_2_O every 30 seconds with a 2 mL/kg decrease in *V*_t_. When PEEP reached 25 cmH_2_O, CPAP at 30 cmH_2_O was used for 30 seconds. Deflation phase	Following period of the study: Patients were randomised into two arms: (1) RM + PEEP at 15 cmH_2_O (*n *= 20) or (2) PEEP alone at 15 cmH_2_O (*n *= 8). A third arm of patients from a preliminary study were analysed: RM only (*n *= 19). PP was used only if PaO_2_/FiO_2 _ratio was < 100 (*n *= 14). The protocol started after 2-hour PP. Data were recorded before and after RM + PEEP (or PEEP only or RM only) at 15, 30, 45 and 60 minutes after the protocol.
Oczenski *et al*., 2005 [44]	Early ARDS (*n *= 15): all extrapulmonary	About 6 mL/kg 460 to 490 mL	18	15	130	29	176	322	CPAP: 50 cmH_2_O for 30 seconds	Following period of the study: After 6-hour PP period, RM was performed. Data were recorded in SP after 6 hours PP and 3, 30 and 180 minutes after RM in SP.
Rival *et al*., 2011 (present study)	Early ARDS (*n *= 16): 12 pulmonary, 4 extrapulmonary	- 540 mL	19	10	98	25	177	218	Extended sigh inflation phase: Pressure levels 30, 35, 40 and 45 cmH_2_O every 30 seconds were used. At 45 cmH_2_O, a 30-second end inspiratory pause was performed. Deflation phase	Following period of the study: 6-hour SP with RM at beginning of SP. Six-hour PP with two RM after 1 hour and 6-hour PP. Measurements taken at beginning of, before and after each RM, and also at end of each ventilation period and 1 hour after end of protocol.

^a^ARDS: acute respiratory distress syndrome; *V*_t_: tidal volume; RR: respiratory rate; PEEP: positive end expiratory pressure; PP: prone position; SP: supine position; RM recruitment manoeuvre; PaO_2_/FiO_2 _ratio, ratio of arterial oxygen partial pressure to fraction of inspired oxygen; Pplat: plateau pressure; CPAP, continuous positive airway pressure.

### Oxygenation efficacy

Our study confirms the efficacy of RM in increasing PaO_2 _in SP and PP. The PaO_2 _improvement was transient in SP. In PP, the efficacy of RM performed after either one hour or six hours was different. First, PaO_2 _did not decrease between the two RMs, and PaO_2 _changes were larger after the second RM. PP and RM may have a combined effect on PaO_2_, and this PaO_2 _improvement would be better if RM were used, probably at different times during PP and especially at the end of PP. A benefit on PaO_2 _was durable one hour after the end of PP. With an extended period of PP (more than 12 hours), the beneficial effect of RM while in PP remains to be demonstrated.

Pelosi *et al*. [43] and Oczenski *et al*. [44] demonstrated the efficacy of such a strategy. In Pelosi *et al*.'s study, sighs were used for one hour after two hours of PP. A positive PaO_2 _variation was found in SP and PP. In SP after RM, PaO_2 _returned to the baseline, whereas in PP, PaO_2 _remained higher than the baseline. In Oczenski *et al*.'s study, extended sigh was used at the end of the PP period, with a persistent increase in oxygenation while the patient was turned supine three hours later. Lim *et al*. [45] showed, first, with an extended sigh, an improvement in PaO_2 _in PP that was lower than in SP, and, second, a PEEP increase after RM prevented the after-RM decrease in PaO_2_/FiO_2 _ratio. The differences between oxygenation responses in SP and PP may be explained by two factors: Only the patients in the most severe condition with a PaO_2_/FiO_2 _ratio < 100 were turned prone in the PP group, and the basic ventilation was delivered with an 8 mL/kg *V*_t_, which could have limited the extent of the effect of the RM [45].

### Recruitment manoeuvre strategy

RM has been studied in experimental models and in clinical studies. An equivalent or superior efficacy of sigh or extended sigh has been demonstrated compared to continuous positive airway pressure (CPAP). In general, a 40 to 50 cmH_2_O peak alveolar pressure is sufficient for lung recruitment [46,47]. The different RMs used in PP are summarized in Table 4 and included sigh, extended sigh and CPAP. They demonstrated a positive effect on alveolar recruitment and oxygenation in SP or PP. In our study, we practiced a RM using pressure control mode, and pressure was progressively increased in steps. The maximum pressure used was 45 cmH_2_O. Compared with RMs described in literature, our method presents some sufficient features to open lung [37,48] with a gradual increase of airway pressure during sufficient time to induce progressive alveolar recruitment and more homogeneous distribution of pressure throughout lung parenchyma. PEEP probably may be increased to stabilize alveolar recruitment and PaO_2 _in SP.

### Respiratory mechanics

In the present study, plateau pressures and PaCO_2 _decreased throughout the PP period and after each RM. PaCO_2 _decreased from 39 mmHg to 36.4 mmHg, and plateau pressure decreased from 24.6 cmH_2_O to 23 cmH_2_O. These results indirectly suggest changes in compliance and alveolar recruitment. Pelosi *et al*. [43] confirmed the benefit of such a ventilatory strategy: In their study, PaCO_2 _showed a decreasing pattern and end expiratory lung volume in PP was higher after RM than it was in SP (277 ± 198 mL vs. 68 ± 83 mL). Compliance followed the same improvement [43].

### Complications

In our study, the protocol had to be interrupted once for arrhythmia and once for bronchoconstriction. Transient hypotension was noted, but MAP remained normal at the end of RM. In a systematic RM review, hypotension (12%) and desaturation (9%) were the most common adverse events. Serious adverse events (barotrauma and arrhythmia) were uncommon [49]. In an experimental model, a decrease in cardiac output was observed [50]. Nielsen *et al*. [51] tested the impact of RM in hypovolemia, normovolemia and hypervolemia. Lung RMs significantly decreased left ventricular end diastolic volume as well as cardiac output during hypovolemia. Caution should be taken, and volemia should be evaluated before starting a RM.

### Methodological considerations and limitations

This study has several limitations. We are unable to argue for the long-lasting effect of the RM and PP combination on PaO_2 _and the benefit of such a strategy performed in all early ALI/ARDS groups. These questions require the enrolment of patients in a crossover study and follow-up of PaO_2 _while the patient is returned to SP. Such a study remains to be done. However, the response with regard to PaO_2 _is quite substantial and already has clinical significance. Because of the relatively small number of patients in our study, we were unable to sort patients according to the type of ARDS (lobar, patchy or diffuse ARDS).

The mechanisms of PaO_2 _improvement cannot be emphasized in our study. With the observed change in plateau pressure for a given *V*_t_, an increase in compliance and an improvement in residual capacity are likely. It would be interesting to measure alveolar recruitment and compliance. As the RM was considered part of daily care, Swan-Ganz catheterisation and cardiac ultrasonography were not systematically performed during the procedure. We do not have the data to analyse the transient haemodynamic instability which occurred during some RMs.

## Conclusions

In clinical practice, and when RM may be used to improve PaO_2 _and decrease VILI, RM may be useful during PP and probably needs to be performed when the patient has been in PP for some time to obtain a full response. Whether a better response is obtained after a longer period of time in PP remains to be demonstrated. The pressure control mode used in our study was as efficient as other methods. However, the place of this strategy needs to be determined in ARDS patients who fail to respond to usual treatment so as not to delay the use of rescue treatments such as extracorporeal membrane oxygenation.

## Key messages

• RM can be used in SP or PP to improve oxygenation.

• A pressure control mode was as efficient as other RMs.

• A probable combined effect on oxygenation exists between PP and RM.

• The combination of PP and RM may be assessed several times, preferably when the patient has been in PP for a few hours.

• No significant side effects were encountered in our study.

## Abbreviations

ALI: acute lung injury; ARDS: acute respiratory distress syndrome; CPAP: continuous positive airway pressure; FiO_2_: fraction of inspired oxygen; MAP: mean arterial pressure; PaO_2_: arterial oxygen partial pressure; PaO_2_/FiO_2 _ratio: ratio of arterial oxygen partial pressure to fraction of inspired oxygen; PaCO_2_: arterial carbon dioxide partial pressure; Paw: peak airway pressure; PEEP: positive end expiratory pressure; PIP: peak inspiratory pressure; PP: prone position; Pplat: plateau pressure; RM: recruitment manoeuvre; RR: respiratory rate; SAPS II: Simplified Acute Physiology Score II; SP: supine position; *V*_t_: tidal volume.

## Competing interests

The authors declare that they have no competing interests.

## Authors' contributions

GR and GC contributed to study conception and design. GR, GC, JCN, EB and CP contributed to patient recruitment into the study. GR contributed to the acquisition of data. NF contributed to the statistical analysis. All investigators commented on, critically revised and read and approved the final manuscript.
